# The Late Prince Francis of Teck and the Middlesex Hospital

**Published:** 1910-11-05

**Authors:** 


### THE LATE PRINCE FRANCIS OF TECK AND THE MIDDLESEX HOSPITAL.

AN extraordinary meeting of Governors of the Middlesex Hospital was held on Friday, October 28, 1910, under the Presidency of his Grace the Duke of Northumberland, to express the deep sympathy felt by the Governors with the Royal House and the late Prince's family on the loss of the late Chairman of the Board. The meeting was very largely attended, and many letters of regret at inability to be present were received.

The Chaplain (the Rev. H. E. Gunton) offered up prayer, and then the Chairman addressed the meeting.

It was, said his Grace, his melancholy duty to propose a vote of condolence on the death of the late Chairman of the hospital; and he reminded the meeting that the last time he presided at a meeting of Governors was a similarly solemn occasion, when they had to deplore the demise of our late Sovereign. Though the loss of Prince Francis was nationally less important, it was, perhaps, as keenly felt by those interested in the hospital, for the late Chairman had so keenly identified himself with the interests of the Middlesex. It was unnecessary to dilate upon the great claims of the Prince to the respect of the nation; he had made a mark in the public service, he had distinguished himself in war, and had shown himself willing to take upon himself the responsibilities of a distinguished member of a great nation. It was only some seven years ago that he began to identify himself with the hospital; yet in that brief time he had rendered services to the hospital which few who had occupied a similar position had ever surpassed. (Hear, hear.) It was only in the spring of this year, after holding other offices, first as a member of the Board and then as Deputy-Chairman, that Prince Francis became Chairman, and in that time he had shown so much energy and so much wise forethought in his conduct of the hospital's affairs, that they felt, in a peculiar manner, the loss due to his death. It must be remembered that there was a heavy debt upon the hospital, but that debt, owing to the Prince's exertions, had been cleared away. It was unnecessary to remind the company that Prince Francis occupied a social position which enabled him to bring his influence to bear upon the interests of the hospital in a wider circle than it was given to most men to influence; but he certainly devoted that influence in the best possible manner—namely, to the relief of distress and the wise consolidation of such a great institution upon a sound and proper basis. He stimulated charity in others, and perhaps the best instance of that was when, last summer, the new wing of the hospital in connection with the Cancer Ward was instituted. All knew how prominent a part he took in that work, and how admirably on that occasion he spoke for the hospital. As was evident from the letter he wrote to the *Times*, he had further efforts in view to place the hospital not only out of debt, but out of danger of ever being in debt again. His Grace trusted that the intentions which the late Prince had in view would still be carried out, for he was sure that those present could not better testify their respect to his memory and their sense of the value of his services. (Hear, hear.) But while remembering how much the hospital was indebted to him, it ought not to be forgotten how very serious had been the loss to the Royal Family, and especially to the Queen. He was sure his hearers would agree that the following humble address of condolence to their Majesties should be sent :—

To their Most Gracious Majesties the King and Queen.

The humble Address of the President, Vice-Presidents, Treasurers, Weekly Board, Medical Staff, Governors, and Officials of the Middlesex Hospital.

May it please your Majesties,

We, your Majesties' dutiful and loyal subjects, humbly venture to approach your Royal Presence to offer our respectful sympathy, and to express our heartful sorrow at the lamented death of Major his late Serene Highness the Prince Francis of Teck.

We wish to record our deep sense of gratitude for the invaluable services which his late Serene Highness rendered to the hospital, first as a member of the Weekly Board, then as a Vice-President and Deputy-Chairman, and, finally, as Chairman.

The earnestness and single-minded devotion with which his late Serene Highness applied himself to the furtherance of the hospital's welfare, and the zeal and energy he displayed in acquainting himself with the details of administration compelled alike our admiration and respect.

It is largely to his late Serene Highness's initiative and performance that the hospital owes its improved reputation for efficiency and its increased power for good. At a time of crisis in the hospital's history he changed anxiety for its future into confidence and hope, and the successful completion of the task he set himself will stand out as the crowning incident of his well-spent efforts on behalf of the suffering poor.

By his sympathy and kindness towards those with whom he came into contact he endeared himself to all, and the regret which is felt at his premature death is general, sincere, and deep.

Sir Kingston Fowler, M.D., K.C.V.O., in seconding the resolution, said their Majesties were well acquainted with the great work being done by the hospital, a work which met with their complete approval. Their Majesties knew the great affection which all at the hospital felt for Prince Francis, whose great characteristic was his invariable amiability. He felt sure their Majesties would receive with extreme pleasure the indication of the sympathy of all those who were associated in the work of the hospital. It would always be to him a matter of satisfaction that it was on his suggestion that Prince Francis first came to the hospital, and it would be a subject of the greatest grief that just when the Prince had begun with so much success the great work there he should have been cut off by the hand of death.

The resolution was carried in silence, the company standing. The Chairman next proposed the following resolution: "That the President, Vice-Presidents, Treasurers, Weekly Board of Governors, Medical Staff, Governors and Officials of the Middlesex Hospital desire respectfully to express their deep sympathy with his Serene Highness the Duke of Teck and the members of his Serene Highness's family in the sad loss which they have sustained by the death of Major his late Serene Highness the Prince Francis of Teck. They wished to place on record their most grateful appreciation of the valuable services his late Serene Highness rendered to the hospital, first as a member of the Weekly Board of Governors, then as a Vice-President and Deputy Chairman, and finally as Chairman. His late Serene Highness exerted all his talents and unsparingly devoted his time to the furtherance of the hospital's welfare, and the fruits of his labours in the cause of suffering humanity will remain as a lasting memorial to his name, which is revered and honoured by all who had the privilege of being associated with him in his work.—Northumberland, President."

Sir A. Pearce Gould, M.S., K.C.V.O., in seconding, said the address was in the name of, among others, the medical staff, and he could speak for all his colleagues when he said that they wished that word on this occasion to have a far wider meaning than ordinarily so as to include all the students in the medical school, who recognised in the Prince a very great friend. Reference had been made to all the late Prince's talents, which were unsparingly devoted to the hospital. He could not help alluding to the variety of those talents, and how ungrudgingly they were devoted to that single object. Reference had been made to his exalted position, and none would forget his splendid presence and graceful courtesy of manner. The courage with which he faced the great task of clearing off that heavy debt, the patient persistence with which he applied himself to it day by day, as well as the humility and dignity with which he undertook every duty in connection with his high position at the institution, would not readily pass away from the memory of those who knew of it. There was a tendency on an occasion like the present to sit with folded hands and think only of one's great loss. If that were done on this occasion great indeed would be the loss; but if, on the other hand, all the friends of the Middlesex Hospital would but imitate what were called the commoner virtues of his late Serene Highness, although they were really none too common, and devote themselves with equal care and persistence and determination day by day, the lesson which the departed Prince had taught would not be lost, and the history of the hospital would show that his influence had long remained. (Hear, hear.)

This resolution was carried in a similarly impressive manner.

The Chairman was cordially thanked for having occupied his post, and for the appropriate words in which he had clothed the sentiments which all felt. The resolution was proposed by the Hon. C. G. Gathorne-Hardy and seconded by the Hon. G. W. Spencer Lyttelton.

In acknowledging the compliment, the Chairman expressed his gratification at the presence of such a large number of friends of the hospital, a gratification which would be echoed by the Royal recipients of the addresses.

